# Diagnostic Efficacy and Correlation of Intravoxel Incoherent Motion (IVIM) and Contrast-Enhanced (CE) MRI Perfusion Parameters in Oncology Imaging: A Systematic Review and Meta-Analysis

**DOI:** 10.1155/ijbi/3621023

**Published:** 2025-11-18

**Authors:** Abhijith S., Rajagopal Kadavigere, Priya P. S., Dharmesh Singh, Priyanka, Tancia Pires, Dileep Kumar, Saikiran Pendem

**Affiliations:** ^1^Department of Medical Imaging Technology, Manipal College of Health Professions, Manipal Academy of Higher Education, Manipal, Karnataka 576104, India; ^2^Department of Radiodiagnosis and Imaging, Kasturba Medical College, Manipal Academy of Higher Education, Manipal, Karnataka 576104, India; ^3^Central Research Institute-Global Scientific Collaboration, United Imaging Healthcare, Shanghai, China

**Keywords:** dynamic contrast-enhanced MRI, dynamic susceptibility contrast MRI, intravoxel incoherent motion, oncology, perfusion MRI

## Abstract

**Background:**

Intravoxel incoherent motion (IVIM) magnetic resonance imaging (MRI) is a noncontrast technique estimating diffusion and perfusion parameters via multiple *b*-values, essential for oncology imaging. However, there is limited collective evidence regarding the efficacy of IVIM in oncology imaging compared to contrast-enhanced (CE) MRI perfusion techniques. This systematic review and meta-analysis compared IVIM's diagnostic accuracy and correlation with CE MRI perfusion techniques.

**Methods:**

Following PRISMA guidelines (PROSPERO-registered), a literature search across five databases (PubMed, Scopus, Embase, Web of Science, and Cochrane Library) was conducted. Diagnostic metrics, including AUC, sensitivity, specificity, and correlation coefficients, were analyzed using a random-effects model, with heterogeneity and publication bias assessed via *I*^2^ statistics and Egger's test.

**Results:**

Eighteen studies on breast, rectal, and brain cancers were analyzed. For breast cancer, IVIM showed 83.50% sensitivity and 81.24% specificity compared to dynamic contrast-enhanced (DCE) MRI's 88.04% sensitivity and 65.98% specificity. In rectal cancer, IVIM achieved 70.9% sensitivity and 56.2% specificity, outperforming DCE MRI's 58.11% sensitivity and 72.49% specificity. For gliomas, IVIM demonstrated 92.27% sensitivity and 74.06% specificity compared to dynamic susceptibility contrast (DSC) MRI's 95.71% sensitivity and 92.91% specificity. Correlations between IVIM and CE parameters were weak to moderate.

**Conclusion:**

IVIM demonstrated equal or superior diagnostic performance to CE MRI in breast cancer, rectal cancer, and gliomas, offering a noncontrast alternative. However, unclear parameter correlations warrant future studies focusing on IVIM protocol optimization based on perfusion regimes.

## 1. Introduction

Magnetic resonance imaging (MRI) is the modality of choice for oncology imaging due to its superior soft tissue contrast and nonionizing radiation [1–4]. To enhance lesion characterization and measure the perfusion parameters, contrast-enhanced (CE) perfusion techniques such as dynamic contrast-enhanced (DCE) imaging and dynamic susceptibility contrast (DSC) imaging play a crucial role [5, 6]. DCE uses a T1-weighted sequence to capture changes in signal intensity as a gadolinium-based contrast agent (GBCA) circulates through tissue. The increase in signal intensity reflects the concentration of the contrast agent, which enables the calculation of perfusion metrics like Ktrans (volume transfer constant), ve (extracellular–extravascular volume fraction), and vp (plasma volume fraction), which provide insights into tissue vascularity, permeability, and perfusion, respectively [7]. DSC uses T2∗-weighted sequences to monitor signal loss caused by susceptibility effects from a bolus injection of GBCAs. As the agent passes through the microvasculature, it induces changes in the magnetic susceptibility of tissues, leading to a signal drop. Perfusion metrics obtained from DSC-MRI include BV (blood volume), BF (blood flow), TTP (time to peak), and MTT (mean transit time) [8].

However, GBCAs used in CE imaging carry risks, including gadolinium deposition in tissues like the brain with uncertain long-term effects and nephrogenic systemic fibrosis (NSF) in patients with severe renal impairment (eGFR < 30 mL/min/1.73m^2^). GBCAs can also trigger hypersensitivity reactions, especially in individuals with allergies. Their use requires cautious risk–benefit evaluation in vulnerable populations, such as those with renal dysfunction and pregnant or breastfeeding women [9, 10].

In addition to CE imaging, diffusion-weighted imaging (DWI) significantly differentiates malignant from benign lesions by estimating cellularity and vascularity [11, 12]. The concept of intravoxel incoherent motion (IVIM) MRI was introduced by Le Bihan et al. [13], which separates diffusion and microvascular perfusion effects in tissues without using contrast agents. It leverages the signal decay in DWI at different *b*-values to capture both molecular water diffusion and perfusion-related pseudodiffusion in capillaries. The critical parameters obtained from IVIM include the true diffusion coefficient (D), reflecting pure tissue diffusion; the pseudodiffusion coefficient (D∗), representing perfusion-related diffusion; and the perfusion fraction (f), which quantifies the proportion of signal attributed to BF [14].

IVIM obtains perfusion parameters without using contrast agents; hence, it may replace CE perfusion, which is beneficial for patients with renal impairment and who need frequent follow-up scans [14]. IVIM also provides diffusion parameters, which may eliminate the need for routine DWI scans, potentially shortening overall scan time.

The literature search identified a lack of comprehensive evidence evaluating the diagnostic accuracy of tumor characterization and the correlation of perfusion metrics between IVIM and CE. To date, no prior meta-analysis has systematically compared these two techniques across multiple oncology imaging studies. Therefore, our systematic review and meta-analysis aimed to fill this gap by comparing the diagnostic accuracy between IVIM and CE perfusion parameters and analyzing the correlation between the parameters of both techniques.

Therefore, our systematic review and meta-analysis investigated perfusion parameters' diagnostic accuracy and correlation between IVIM and CE perfusion parameters in oncology imaging.

## 2. Methodology

### 2.1. Study Registration

This systematic review and meta-analysis followed the guidelines outlined in the Preferred Reporting Items for Systematic Reviews and Meta-Analyses (PRISMA) [15]. The PRISMA checklist used for the meta-analysis can be found in Supporting Information 1: File S1. The protocol of this review is registered under PROSPERO (International Prospective Register of Systematic Reviews, Review ID: CRD42024568365). A preliminary version of this study was previously made available as a preprint on Research Square [16].

### 2.2. Databases and Search Strategies

A literature search was conducted among the following databases: PubMed, Scopus, Embase, Web of Science, and Cochrane Library. The search terms used for the literature search were as follows: “Meningioma”, “Glioma”, “Brain Neoplasms”, “Breast Neoplasms”, “Breast Cancer Lymphedema,” “Carcinoma, Hepatocellular”, “Liver Neoplasms”, “Carcinoma, Renal Cell”, “Renal Neoplasms”, “Rectal Neoplasms”, “IVIM”, “Intravoxel incoherent motion”, “Multi b value”, “biexponential” “DWI”, “Diffusion magnetic resonance imaging”, “Dynamic contrast enhanced”, “DCE”, “Dynamic susceptibility contrast,” “DSC”, “Contrast enhanced MR”, “Multiparametric”, “Efficacy”, “Sensitivity”, “Specificity”, “Correlation”, “Effectiveness”. For each database, the search strategies used are provided in Supporting Information 2: File S2.

### 2.3. Selection Criteria

The review employed the participants, intervention, comparison, and outcomes (PICO) methodology to select articles, as detailed in Table 1.

*Inclusion criteria*: The study included articles that compared the efficacy of IVIM with CE perfusion MRI for characterizing histopathology-confirmed lesions in oncology imaging. Studies that correlated perfusion metrics obtained from IVIM and CE MRI were also included.

*Exclusion criteria*: Articles were excluded if they did not report receiver operating characteristic (ROC) curve analysis or correlation coefficients between both techniques. Other articles, including meta-analyses, reviews, or surveys, were excluded.

### 2.4. Data Extraction

Search strategies for the literature review were conducted independently by two reviewers. Initially, titles and abstracts were screened for the eligibility criteria, and eligible articles were sought for full-text availability. The eligible articles were extracted with the following parameters: title, author, year of publication, country, scanner and field strength, *b*-values, sample size, IVIM and CE perfusion metrics, sensitivity, specificity, area under the curve (AUC), and correlation coefficients. Any discrepancies in the results of the two reviewers were discussed and resolved through consensus.

### 2.5. Quality Assessment

The quality of the studies and the risk of bias assessment were evaluated using the Diagnostic Study Appraisal Worksheet created by the Centre for Evidence-Based Medicine at the University of Oxford [17]. The screening was performed by two reviewers independently, and any discrepancies in the decision were resolved by discussion.

### 2.6. Statistical Analysis

A meta-analysis was conducted to evaluate the diagnostic accuracy of IVIM compared to DSC/DCE using pooled estimates of sensitivity, specificity, and AUC. Pooled estimates and their 95% confidence intervals (CIs) for sensitivity and specificity were calculated using a random-effects model with restricted maximum likelihood (REML) estimation to account for between-study heterogeneity. Summary receiver operating characteristic (SROC) analyses were conducted to visualize diagnostic performance. These included confidence region plots and weighted crosshair plots, displaying sensitivity, specificity, and CIs for individual studies and pooled results. Publication bias was assessed using Egger's regression test, performed separately for the AUC, sensitivity, and specificity metrics. Pearson's correlation coefficients (*r*) between IVIM and DSC/DCE parameters were obtained to assess correlations between diagnostic parameters. Fisher's *Z* transformation was applied to stabilize variance before pooling estimates using a random-effects model. Forest plots were used to display pooled correlation estimates and their CIs, while funnel plots were constructed to evaluate potential publication bias and the distribution of effect sizes. The results were subsequently back-transformed to Pearson's *r* for interpretability. Correlation coefficients were scaled *weak* (< 0.39), *moderate* (0.4–0.59), and *strong* (≥ 0.6).

## 3. Result

**Figure 1:**
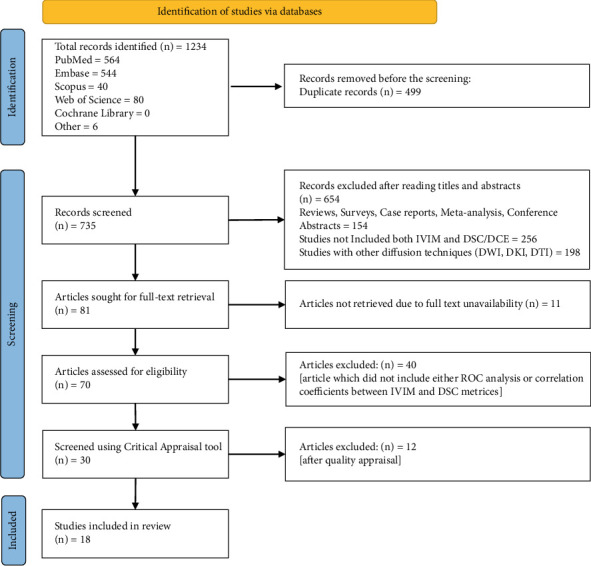
PRISMA flow chart.

A total of 1234 articles were identified across various databases. Of these, 499 were duplicates and were removed. The remaining 735 articles were screened based on title and abstract according to the study's inclusion criteria, leading to the exclusion of 654 articles. Subsequently, 81 articles were selected for full-text retrieval, but 11 were unavailable. The 70 available full texts were screened for inclusion, resulting in the exclusion of 41. Finally, 29 articles underwent critical appraisal, and 18 were included in the review [18–35].  Figure 1 outlines the PRISMA flow diagram used for the study selection process for the review.

### 3.1. Study Characteristics and Quality Assessment

In the final analysis, 18 studies were included, with seven on the brain, six on the breast, and five on the rectum. A comprehensive overview of the baseline characteristics for all studies is provided in Table 2. Quality assessment was conducted across three key domains: study validity, study outcomes, and the applicability of findings. Each domain was rated on a scale from 0 to 3. The total score for each study was divided by the maximum possible score to calculate a percentage. Based on these percentages, the studies were classified into three categories: *poor* (< 35%), *average* (35%–69%), and *good quality* (> 70%). Among the 30 studies evaluated, 10 were categorized as poor, seven as average, and 13 as good quality. The quality assessment score of each study included can be found in Supporting Information 3: File S3.

The literature search included studies involving brain, breast, liver, renal, and rectal cancers. After screening, only studies on brain, breast, and rectal cancers met the selection criteria and were included in this review. The meta-analysis of diagnostic performance in brain tumors was limited to gliomas, as no other tumor types fulfilled the selection criteria.

### 3.2. Diagnostic Performance of IVIM and DCE in Breast Cancer

**Figure 2:**
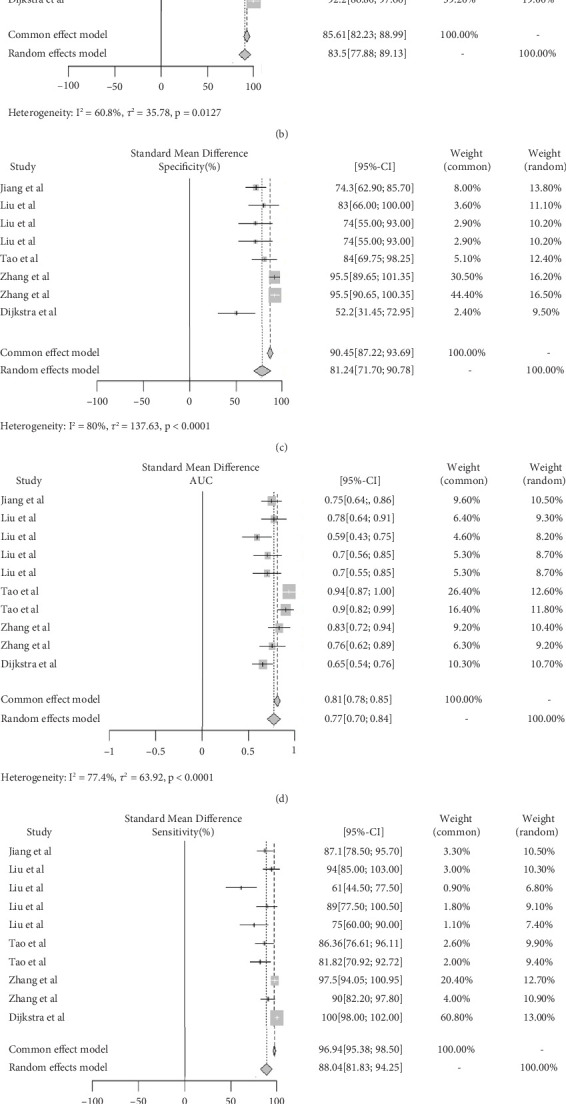
Diagnostic performance of IVIM and DCE for breast cancer. (a–c) The AUC, sensitivity, and specificity of IVIM parameters, respectively. (d–f) The AUC, sensitivity, and specificity of DCE parameters, respectively.

**Figure 3:**
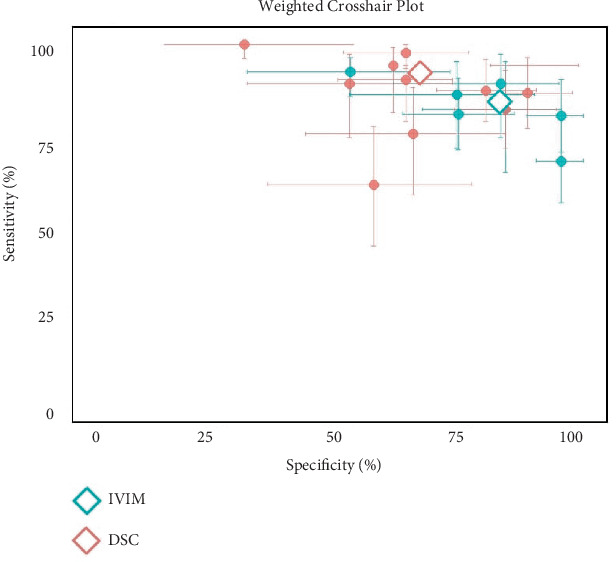
Weighted crosshair plot (SROC) for diagnostic performance of IVIM and DCE in breast cancer.

The meta-analysis included a total of 231 breast lesions for IVIM and DCE. For IVIM, the pooled AUC was 0.84 (95% CI: 0.80–0.88), with sensitivity of 83.50% (95% CI: 77.88%–89.13%) and specificity of 81.24% (95% CI: 71.70%–90.78%). The *I*^2^ was 3.5%, 60.8%, and 80% for AUC, sensitivity, and specificity, respectively (Figure 2). Egger's test for funnel plot asymmetry for publication bias resulted in *t* = −4.21, *p* = 0.006 [18–20, 22, 23]. For DCE, the pooled AUC was 0.77 (95% CI: 0.70–0.84), with sensitivity of 88.04% (95% CI: 81.83%–94.25%) and specificity of 65.98% (95% CI: 55.66%–76.30%). The *I*^2^ was 77.4%, 84.4%, and 82.8% for AUC, sensitivity, and specificity, respectively (Figure 2). Egger's test for funnel plot asymmetry for publication bias resulted in *t* = −4.54, *p* = 0.002 [17–19, 21, 22].  Figure 3 presents the weighted crosshair plots for these analyses, visually summarizing diagnostic performance across studies.

### 3.3. Diagnostic Performance of IVIM and DCE in Rectal Cancer

**Figure 4:**
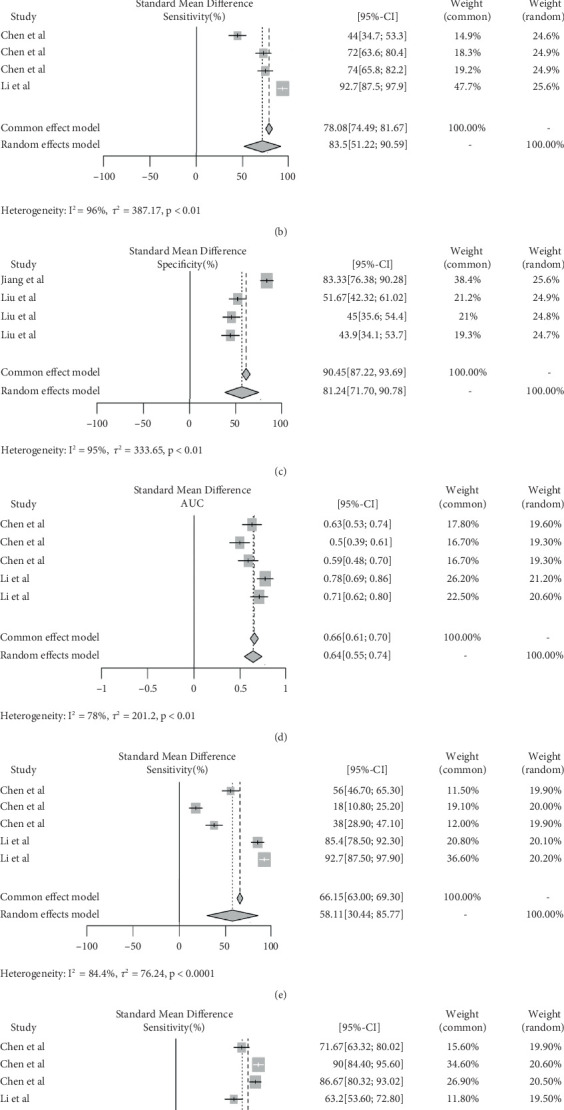
Diagnostic performance of IVIM and DCE for rectal cancer. (a–c) The AUC, sensitivity, and specificity of IVIM parameters, respectively. (d–f) The AUC, sensitivity, and specificity of DCE parameters, respectively.

**Figure 5:**
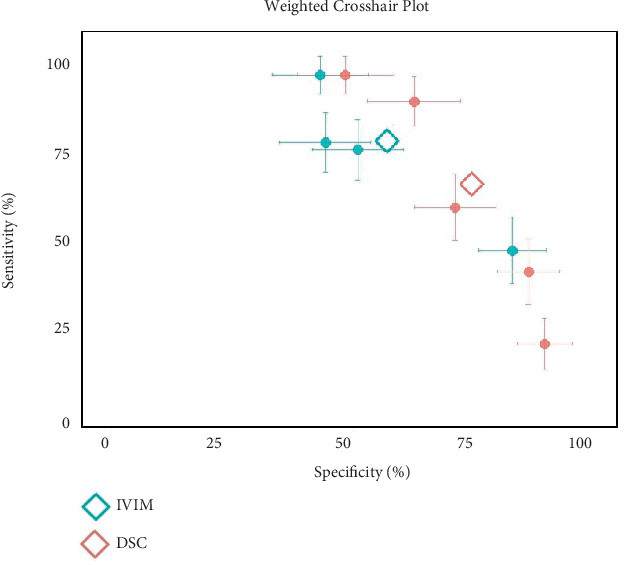
Weighted crosshair plot (SROC) for diagnostic performance of IVIM and DCE in rectal cancer.

The meta-analysis included a total of 208 lesions. For IVIM, a pooled AUC of 0.62 (95% CI: 0.53–0.71), sensitivity of 70.9% (95% CI: 51.22%–90.59%), and specificity of 56.2% (95% CI: 37.75%–74.64%) were observed. The *I*^2^ was 67%, 96%, and 95% for AUC, sensitivity, and specificity, respectively (Figure 4). Egger's test for funnel plot asymmetry indicated significant publication bias for both AUC (*t* = −4.50, *p* = 0.0459) and specificity (*t* = −12.45, *p* = 0.0064), but not for sensitivity (*t* = −3.30, *p* = 0.0809) [25, 26]. The result for DCE was a pooled AUC of 0.64 (95% CI: 0.55–0.74), sensitivity of 58.11% (95% CI: 30.44%–85.77%), and specificity of 72.49% (95% CI: 57.71%–87.26%). The *I*^2^ was 78%, 99%, and 94% for AUC, sensitivity, and specificity, respectively (Figure 4). Egger's test for funnel plot asymmetry indicated no significant publication bias for sensitivity (*t* = −1.49, *p* = 0.2334) and specificity (*t* = −7.28, *p* = 0.0054) but suggested potential bias for other measures (*t* = −8.31, *p* < 0.001) [25, 26].  Figure 5 presents the weighted crosshair plots for these analyses, visually summarizing diagnostic performance across studies.

### 3.4. Diagnostic Performance of IVIM and DSC in Glioma

**Figure 6:**
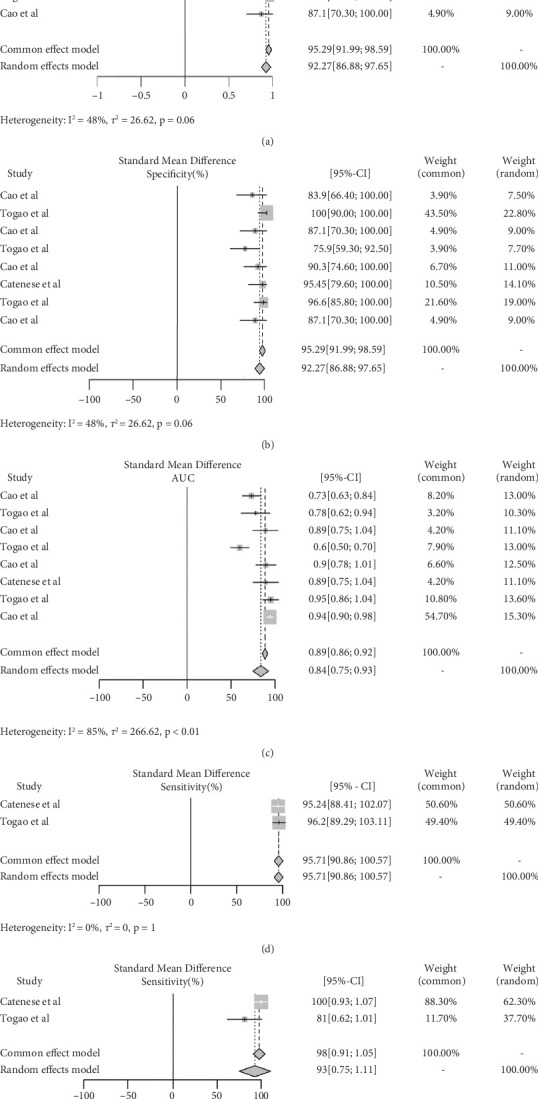
Diagnostic performance of IVIM and DSC for glioma. (a–c) The AUC, sensitivity, and specificity of IVIM parameters, respectively. (d, e) The sensitivity and specificity of DSC parameters, respectively.

**Figure 7:**
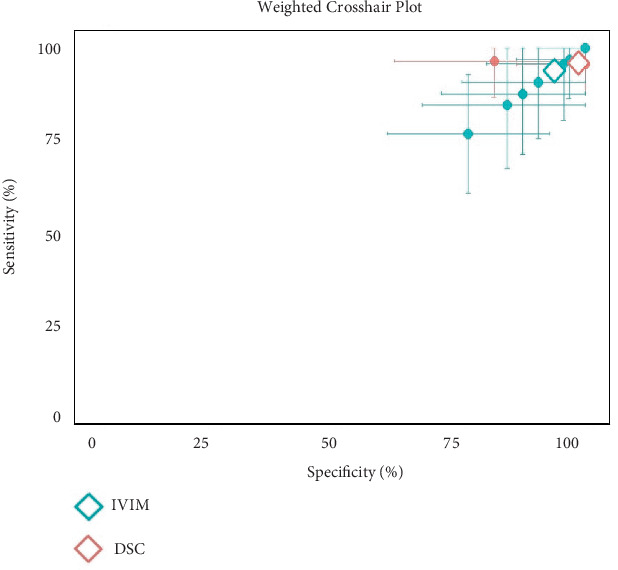
Weighted crosshair plot (SROC) for diagnostic performance of IVIM and DCE in glioma.

The meta-analysis evaluated diagnostic accuracy for 123 and 73 gliomas for IVIM and DSC, respectively. For IVIM, the pooled AUC was 0.84 (95% CI: 0.75–0.93), with a sensitivity of 92.27% (95% CI: 86.88%–97.65%) and specificity of 74.06% (95% CI: 60.51%–87.61%). The heterogeneity index (*I*^2^) was 85%, 48%, and 89% for AUC, sensitivity, and specificity, respectively (Figure 6) Egger's test for funnel plot asymmetry for publication bias resulted in *t* = −1.56, *p* = 0.170 [29, 33, 34]. For DSC, the meta-analysis revealed a pooled sensitivity of 95.71% (95% CI: 90.86%–100%) and specificity of 92.91% (95% CI: 75.06%–100%), with *I*^2^ values of 0% and 68%, respectively (Figure 6) [33, 34].  Figure 7 presents the weighted crosshair plots for these analyses, visually summarizing diagnostic performance across studies.

### 3.5. Correlation Between IVIM and DSC Parameters in Brain

**Figure 8:**
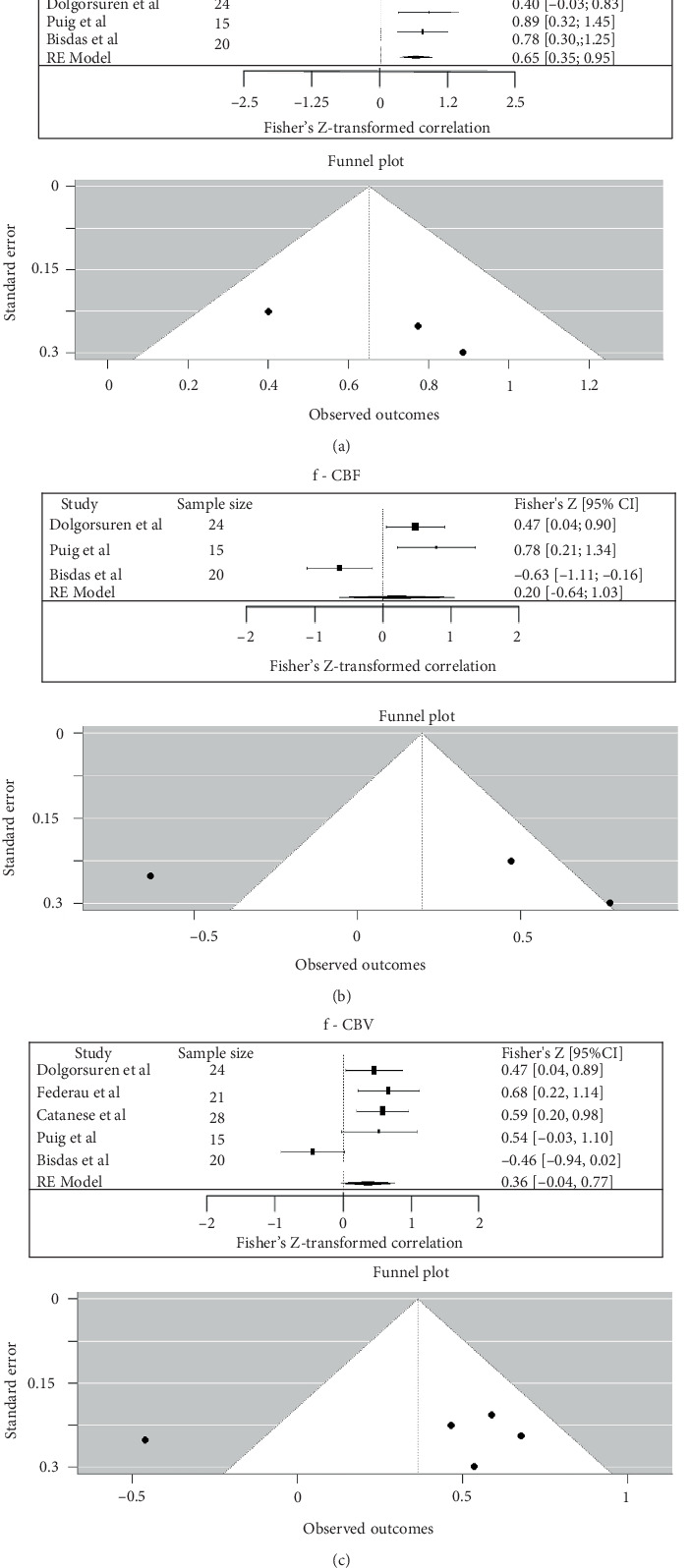
(a–c) Forest plot for meta-analysis of Pearson's correlation coefficients between IVIM and DSC in glioma (D∗—CBF, f—CBF, and f—CBV).

The meta-analysis of correlation coefficients between IVIM and DSC parameters is summarized in Figure 8. Between D∗ and CBF, the pooled Fisher's *Z*-transformed correlation was 0.65 (95% CI: 0.35–0.95), which corresponded to a back-transformed Pearson's *r* value of 0.57 (95% CI: 0.34–0.74) (*I*^2^ = 15.1%). Between f and CBF, the pooled Fisher's *Z*-transformed correlation was 0.20 (95% CI: −0.64 to 1.03), which corresponded to a back-transformed Pearson's *r* value of 0.20 (95% CI: −0.56 to 0.77) (*I*^2^ = 88.69%) [30, 32, 35]. Between f and CBV, the pooled Fisher's *Z*-transformed correlation was 0.36 (95% CI: −0.04 to 0.77), which corresponded to a back-transformed Pearson's *r* value of 0.34 (95% CI: −0.04 to 0.64) (*I*^2^ = 74.34%) [30–32, 34, 35]. The individual study estimates and their CIs were displayed in the forest plot and corresponding funnel plots (Figure 8).

### 3.6. Correlation Between IVIM and DCE Parameters in Rectum

**Figure 9:**
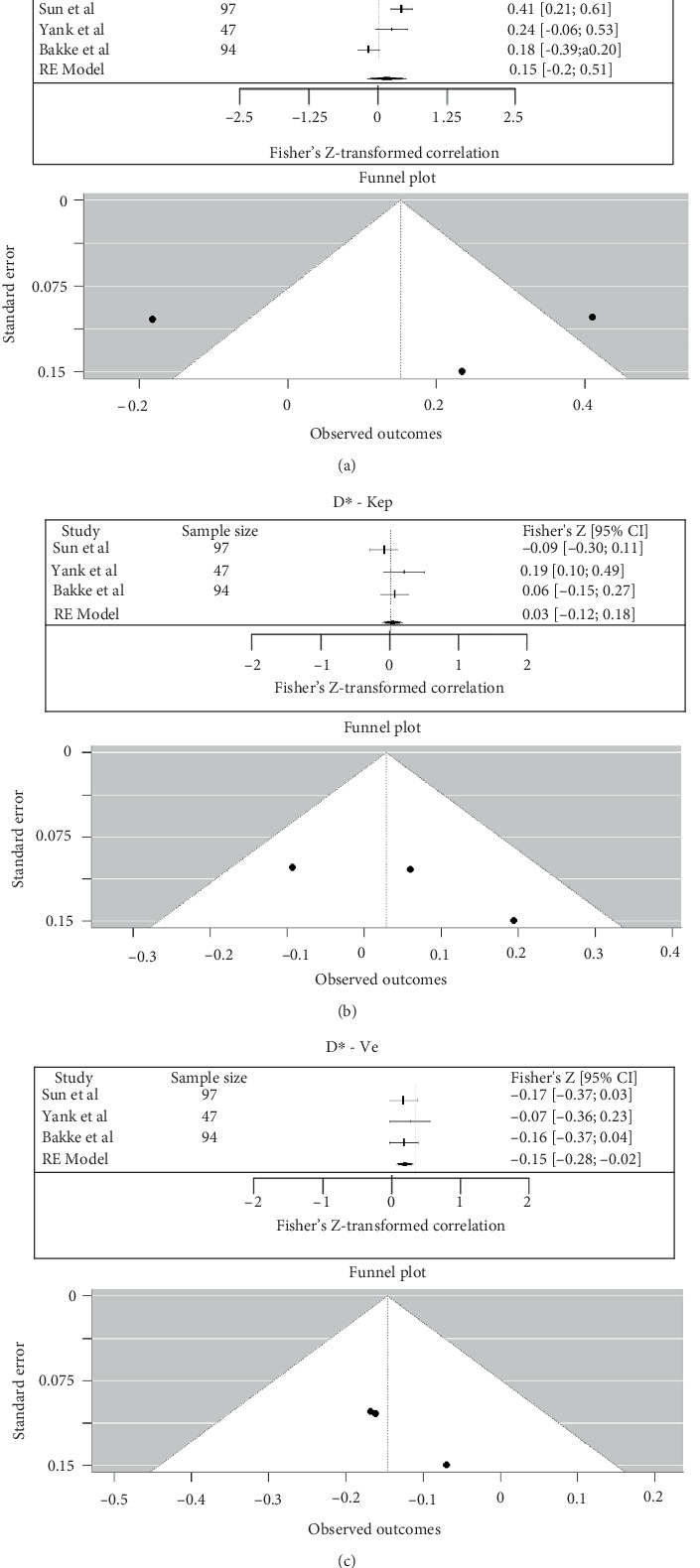
(a–c) Forest plot for meta-analysis of Pearson's correlation coefficients between IVIM and DCE in rectal cancer (D∗—Ktrans, D∗—Kep, and D∗—Ve).

The meta-analysis of correlation coefficients between IVIM and DCE parameters is summarized in Figure 9. Between D∗ and Ktrans, the pooled Fisher's *Z*-transformed correlation was 0.15 (95% CI: −0.20 to 0.51), which corresponded to a back-transformed Pearson's *r* value of 0.15 (95% CI: −0.20 to 0.47) (*I*^2^ = 86.07%). Between D∗ and Kep, the pooled Fisher's *Z*-transformed correlation was 0.03 (95% CI: −0.12 to 0.18), which corresponded to a back-transformed Pearson's *r* value of 0.03 (95% CI: −0.12 to 0.18) (*I*^2^ = 23.1%). Between D∗ and Ve, the pooled Fisher's *Z*-transformed correlation was −0.15 (95% CI: −0.28 to −0.02), which corresponded to a back-transformed Pearson's *r* value of 0.15 (95% CI: −0.27 to −0.02) (*I*^2^ = 0%) (Figure 9) [24, 27, 28].

**Figure 10:**
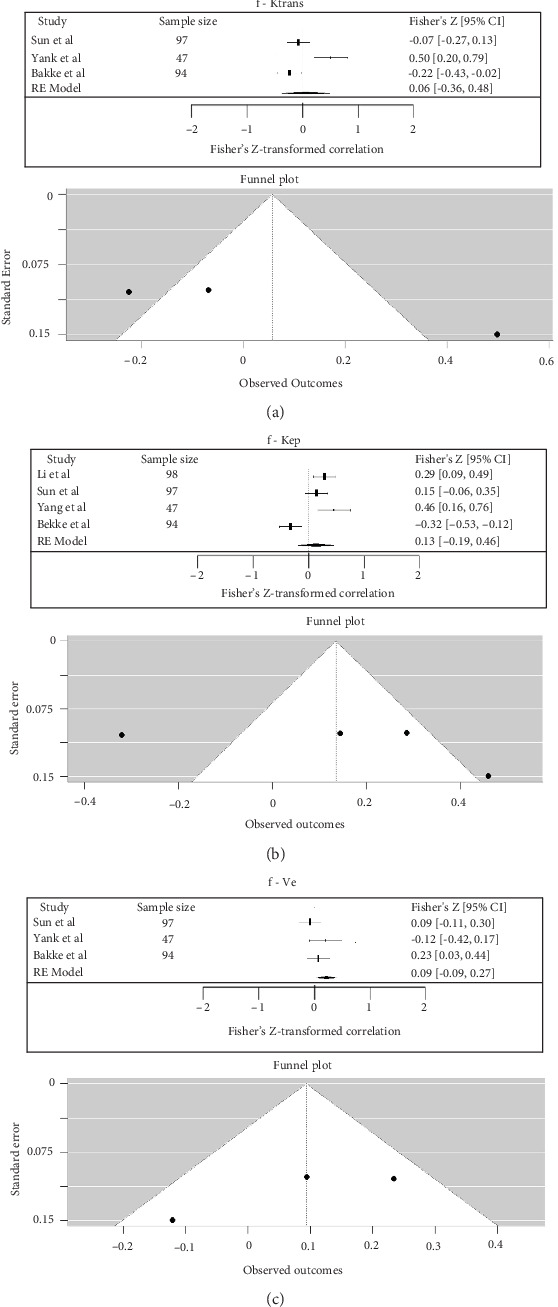
(a–c) Forest plot for meta-analysis of Pearson's correlation coefficients between IVIM and DCE in rectal cancer (f—Ktrans, f—Kep, and f—Ve).

Between f and Ktrans, the pooled Fisher's *Z*-transformed correlation was 0.06 (95% CI: −0.36 to 0.48), which corresponded to a back-transformed Pearson's *r* value of 0.06 (95% CI: −0.34 to 0.45) (*I*^2^ = 89.91%). Between f and Kep, the pooled Fisher's *Z*-transformed correlation was 0.13 (95% CI: −0.19 to 0.46), which corresponded to a back-transformed Pearson's *r* value of 0.13 (95% CI: −0.19 to 0.43) (*I*^2^ = 88.48%). Between f and Ve, the pooled Fisher's *Z*-transformed correlation was 0.09 (95% CI: −0.09 to 0.27), which corresponded to a back-transformed Pearson's *r* value of 0.09 (95% CI: −0.09 to 0.26) (*I*^2^ = 45.89%) (Figure 10) [24, 27, 28].

**Figure 11:**
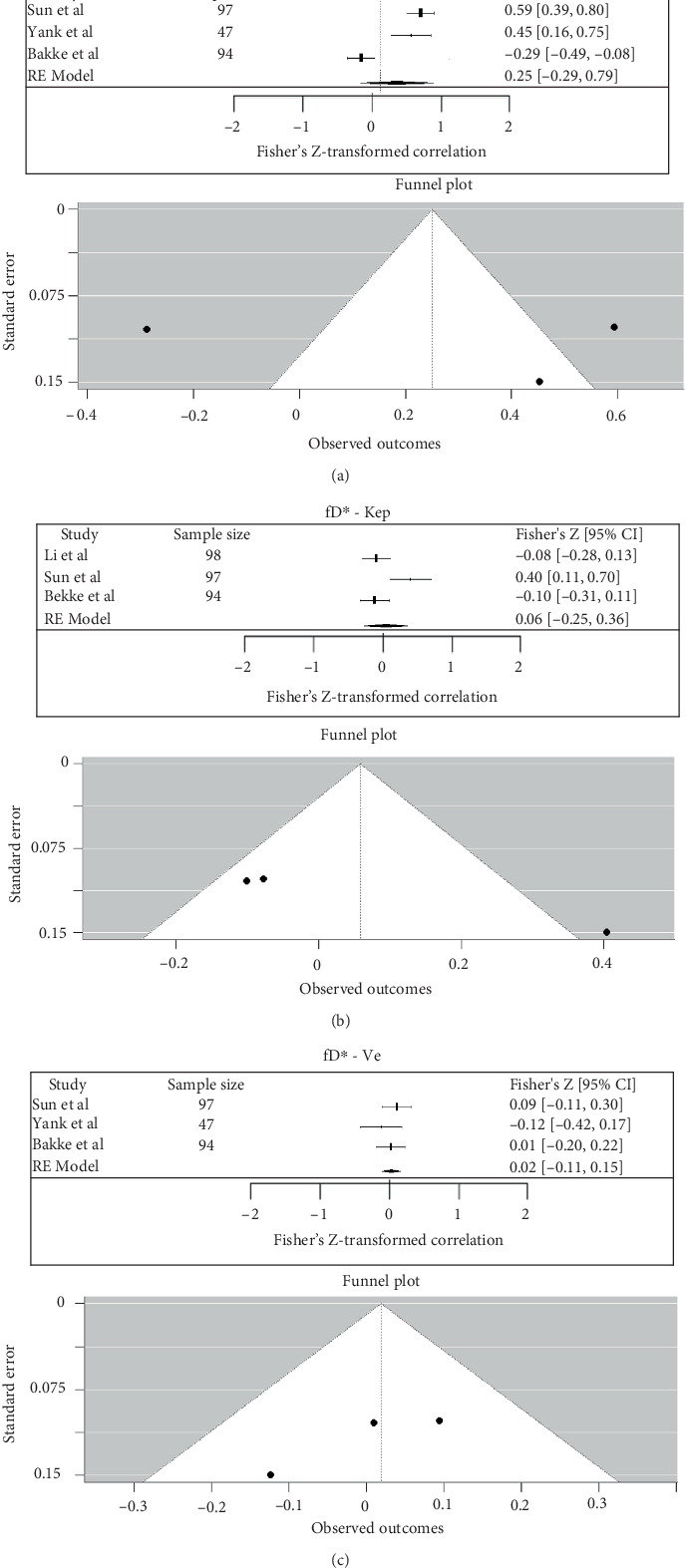
(a–c) Forest plot for meta-analysis of Pearson's correlation coefficients between IVIM and DCE in rectal cancer (fD∗—Ktrans, fD∗—Kep, and fD∗—Ve).

Between fD∗ and Ktrans, the pooled Fisher's Z-transformed correlation was 0.25 (95% CI: −0.29 to 0.79), which corresponded to a back-transformed Pearson's *r* value of 0.24 (95% CI: −0.28 to 0.66) (*I*^2^ = 94.01%). Between fD∗ and Kep, the pooled Fisher's *Z*-transformed correlation was 0.06 (95% CI: −0.25 to 0.36), which corresponded to a back-transformed Pearson's *r* value of 0.06 (95% CI: −0.24 to 0.34) (*I*^2^ = 80.91%) [24, 26–28]. Between D∗ and Ve, the pooled Fisher's *Z*-transformed correlation was 0.02 (95% CI: −0.11 to 0.15), which corresponded to a back-transformed Pearson's *r* value of 0.02 (95% CI: −0.11 to 0.15) (*I*^2^ = 0%) (Figure 11) [24, 27, 28].

## 4. Discussion

Diffusion and perfusion MR is widely used in oncology imaging due to its noninvasive and nonionizing nature for tumor structure, perfusion, and permeability assessment [36, 37]. CE perfusion MRI plays a significant role in functional characterization, whereas diffusion imaging will provide the structural information of the tissue. The IVIM sequence includes lower *b*-values less than 200 s/mm^2^ (sensitive to microvascular perfusion) and higher *b*-values to quantify molecular diffusion, which has gained increasing prominence in oncology imaging [14]. However, there is a notable lack of consolidated evidence regarding the overall efficacy of IVIM compared to CE perfusion MRI in this context. This systematic review and meta-analysis aimed to compare the diagnostic performance of perfusion metrics derived from IVIM with those obtained from CE perfusion MRI.

MRI remains the modality of choice for breast cancer diagnosis and follow-up, with CE perfusion and diffusion imaging playing an important role. This highlights the relevance of assessing the feasibility of IVIM in this context [38, 39]. Present meta-analysis findings suggest that IVIM imaging parameters exhibit strong diagnostic performance for differentiating benign and malignant breast lesions, with high sensitivity and specificity. IVIM demonstrated slightly superior diagnostic accuracy compared to DCE parameters, reinforcing its potential as a valuable tool in clinical practice. While DCE also showed good sensitivity, its specificity was relatively lower, indicating a higher likelihood of false positives. Human epidermal growth factor receptor 2 (HER2)–enriched and triple-negative breast cancers (TNBCs) are aggressive subtypes, often challenging to distinguish [40, 41]. Notably, IVIM has shown promise in differentiating these subtypes, with even greater efficacy when combined with CE perfusion MRI [42, 43].

The meta-analysis results suggest that IVIM and DCE exhibit comparable diagnostic performance in assessing rectal cancer. IVIM demonstrated slightly higher sensitivity, while DCE showed better specificity, highlighting the complementary potential of these techniques. Meyer et al. evaluated IVIM parameters against histological findings and concluded that IVIM parameters effectively diagnose rectal cancer. Notably, D and ADC values could distinguish proliferation status, while f is strongly associated with microvascular density [44]. Other parameters, such as D∗, fD∗, and f, were significantly different across grades of rectal cancer with significant diagnostic efficacy [45–47].

The meta-analysis highlights the diagnostic potential of IVIM and DSC in glioma evaluation, with DSC being recognized for its strong diagnostic performance [48]. However, compared to IVIM, the literature on DSC is relatively limited, particularly in studies assessing a comprehensive range of diagnostic parameters. Additionally, Wang and Dong [49] investigated the prediction of isocitrate dehydrogenase (IDH) mutation status, which offers valuable insights into the biological behavior, treatment approaches, and prognosis of gliomas. Their study concluded that perfusion and diffusion parameters derived from IVIM imaging could predict IDH mutation status with high sensitivity and specificity, especially the perfusion fraction (f). Further extending the application of IVIM, Lu et al. [50] demonstrated its potential in predicting the methylation status of O^6^-methylguanine-DNA-methyltransferase (MGMT) in gliomas, with IVIM parameters showing sensitivity and specificity comparable to or exceeding those of DSC imaging. Also, Puig et al. [32] found that IVIM parameters effectively predict overall survival in patients with glioblastoma.

In addition to diagnostic performance, we also checked for the correlation between IVIM and CE perfusion parameters. Specifically, in the brain, D∗ showed a moderate correlation (0.57) with CBF, and f showed a weak correlation with CBF (0.2) and CBV (0.34), which are the parameters of DSC. The principle behind the perfusion estimation for both techniques is entirely different, which limits the direct comparison of parameters. Furthermore, the proper selection of acquisition parameters is crucial for accurate perfusion estimation. For example, a study by Bisdas et al. demonstrated a positive correlation between IVIM and DSC perfusion parameters in tumors; however, the correlation was weak or even negative in healthy tissue. They suggested that this discrepancy might be due to the low BF regime in healthy brain tissue, which reduces the signal-to-noise ratio (SNR) and affects the reliability of IVIM measurements [35, 51]. This highlights a key limitation of this meta-analysis: The acquisition parameters across all included studies were not uniform. This inconsistency could also explain why some studies demonstrated weak correlations between parameters [52, 53].

The meta-analysis revealed weak and variable correlations between IVIM and DCE parameters in the rectum, with notable heterogeneity across studies. These findings underscore the complexity of the relationship between IVIM and DCE-derived metrics. As discussed earlier, optimizing acquisition parameters is crucial in low-perfusion conditions to achieve a high SNR and ensure that IVIM-derived perfusion estimates align with those obtained from CE perfusion techniques [24, 26–28]. Overall, further detailed comparisons are required to compare with DCE in rectal cancer diagnosis to better understand the relative advantages of IVIM.

### 4.1. Limitations

The limited number of studies for each cancer type restricts the generalizability of our findings. Additionally, only studies on brain (glioma), breast, and rectal cancers were included, highlighting a gap that warrants further investigation. Although we initially aimed to include brain, breast, liver, renal, and rectal cancers, liver and renal cancers were ultimately excluded due to the lack of sufficient eligible studies, which limits the comprehensiveness of our analysis. Variability in imaging protocols, scanner types, and analytical methods across studies may have introduced inconsistencies in the reported diagnostic performance, complicating direct comparisons. For glioma, the number of cases included in the meta-analysis differed between IVIM and DSC techniques due to reporting bias in the selected studies. Although some studies demonstrated correlations between perfusion parameters obtained from both methods, conflicting results were also observed, suggesting the need for further validation. Future research should aim to evaluate the accuracy and reliability of perfusion parameters derived from IVIM to enhance its clinical utility.

## 5. Conclusion

This meta-analysis highlights IVIM as a promising noninvasive alternative to CE perfusion MRI in oncology imaging. IVIM demonstrates comparable diagnostic performance across gliomas, breast, and rectal cancers, with additional advantages in sensitivity for specific tumor characteristics and prognostic markers. However, variability in imaging protocols and nonoptimized acquisition parameters limit direct comparisons with CE perfusion metrics. Future multicenter studies with standardized protocols are essential to validate IVIM as a routine clinical alternative to CE MRI.

## Figures and Tables

**Table 1:** PICO methodology of the study.

**Characteristics**	**Criteria**
Population	Patients diagnosed with histopathology-confirmed brain, breast, liver, renal, and rectal cancers
Intervention	Intravoxel incoherent motion MRI
Comparator	Dynamic contrast-enhanced (DCE)/dynamic susceptibility contrast (DSC) MRI
Outcomes	AUC, sensitivity, specificity, and correlation coefficients of IVIM matrices with contrast-enhanced perfusion metrics

**Table 2:** Study characteristics.

**Sl no.**	**Author and year**	**Part scanned**	**Pathology**	**Scanner specifications**	**Number of lesions**	**Comparator technique used**	**Gold standard**	**Quality assessment score (%)**
*Breast*								
1	Dijkstra et al., 2016 [18]	Breast	Benign vs. malignant lesion	1.5T Magnetom Avanto, Siemens	139	DCE	Histopathology	77.77
2	Jiang et al., 2018 [19]	Breast	Benign vs. malignant lesion	3T Discovery 750, GE	66 (35 benign and 31 malignant)	DCE	Histopathology	77.77
3	Liu et al., 2016 [20]	Breast	Benign vs. malignant lesion	1.5T, Philips	59 (23 benign and 36 malignant)	DCE	Histopathology	100
4	Ma et al., 2017 [21]	Breast	Benign vs. malignant lesion	3T Skyra, Siemens	117 (47 benign and 81 malignant)	DCE	Histopathology	55.55
5	Tao et al., 2019 [22]	Breast	Benign vs. ductal carcinoma	3T Verio, Siemens	47 (22 benign and 25 ductal carcinoma)	DCE	Histopathology	66.66
6	Zhang et al., 2024 [23]	Breast	Benign vs. malignant lesion	3T Ingenia CX, Philips	59 (40 malignant and 22 benign lesions)	DCE	Histopathology	88.88
*Rectal*								
7	Bakke et al., 2019 [24]	Rectal	Rectal cancer	1.5T Achieva, Philips	94	DSC and DCE	Histopathology	55.55
8	Chen et al., 2022 [25]	Rectal	Rectal cancer	3T Prisma, Siemens	110	DCE	Histopathology	77.77
9	Li et al., 2021 [26]	Rectal	Rectal cancer	3T Inter Achieva, Philips	98	DCE	Histopathology	77.77
10	Sun et al., 2019 [27]	Rectal	Rectal cancer	3T Ingenia, Philips	97	DCE	Histopathology	77.77
11	Yang et al., 2019 [28]	Rectal	Rectal cancer	3T Magnetom Veria, Siemens	47	DCE	Histopathology	55.55
*Brain*								
12	Cao et al., 2017 [29]	Brain	Glioma	3T Signa HDxt, GE	50 (19 low and 31 high grade)	DCE	Histopathology	88.88
13	Dolgorsuren et al., 2019 [30]	Brain	Glioma and lymphoma	3T Discovery 750, GE	24	DSC	Histopathology and clinical	55.55
14	Federau et al., 2014 [31]	Brain	Glioma	3T Trio, Verio, or Sykra, Siemens	21 (5 low and 16 high grade)	DSC	Histopathology	66.66
15	Puig et al., 2016 [32]	Brain	Glioma	1.5T Hyroscan Intera, Philips	15	DSC	Histopathology	88.88
16	Togao et al., 2016 [33]	Brain	Glioma	3T Achieva TX, Philips Healthcare	45 (16 low and 29 high grade)	DSC	Histopathology	88.88
17	Catanese et al., 2018 [34]	Brain	Glioma	3T Signa Excite, GE	28 (6 low and 22 high grade)	DSC	Histopathology	88.88
18	Bisdas et al., 2014 [35]	Brain	Glioma	3T Magnetom Verio, Siemens	20	DSC and DCE	Histopathology	44.44

Abbreviations: DCE, dynamic contrast-enhanced; DSC, dynamic susceptibility contrast.

## Data Availability

The datasets used and/or analyzed in the current study are available from the corresponding author upon reasonable request.

## Supporting Information

Supporting Information 2 Search strategies used for each database are supplied in File S2.

Supporting Information 3 The quality assessment score process and scores of each study are in File S3.
