# Supplementary material for: Diagnostic Efficacy and Correlation of Intravoxel Incoherent Motion (IVIM) and Contrast-Enhanced (CE) MRI Perfusion Parameters in Oncology Imaging: A Systematic Review and Meta-Analysis
Source: Int J Biomed Imaging. 2025 Nov 18;2025:3621023. doi: 10.1155/ijbi/3621023 (PMC12646736; doi:10.1155/ijbi/3621023)
Supplement: Supporting Information 2 — Search strategies used for each database are supplied in File S2. [file 3621023.f2.pdf]

Supplementary File 3

1: Critical appraisal of brain studies

Sl No	1	2	3	4	5	6	7	8	9	10	11
**Author**	Bisdas et al	Cao et al	Catanese et al	Dolgorsuren et al	Federau et al	Federau et al	Hellström et al	Pettengill et al	Puig et al	Wang et al	Togao et al
**Critical Appraisal**											
**Step 1: Are the results of the study valid?**											
1. Was the diagnostic test evaluated in a Representative spectrum of patients (like those in whom it would be used in practice)?	1	1	1	1	1	1	1	0	1	1	1
2.Was the reference standard applied regardless of the index test result?	0	1	1	1	1	1	1	1	1	1	1
3. Was there an independent, blind comparison between the index test and an appropriate reference ('gold') standard of diagnosis?	0	1	0	0	0	0	0	0	1	1	1
**Step 2: What were the results?**											
4.Are test characteristics presented?	0	3	3	0	0	2	1	0	3	3	3
**Step 3: Applicability of the results**											
5. Were the methods for performing the test described in sufficient detail to permit replication?	3	2	3	3	1	2	0	0	2	3	2
**Total Score**	**4**	**8**	**8**	**5**	**3**	**6**	**3**	**1**	**8**	**9**	**8**
**Quality (%)**	**44.44444444**	**88.88888889**	**88.88888889**	**55.55555556**	**33.33333333**	**66.66666667**	**33.33333333**	**11.11111111**	**88.88888889**	**100**	**88.88888889**

2: Critical Appraisal of Breast Studies

**Sl No**	**1**	**2**	**3**	**4**	**5**	**6**	**7**	**8**	**9**	**10**	**11**	**12**
**Author**	Almutlaq et al	Dijkstra et al	Jiang et al	Liu et al	Ma et al	Sun et al	Suo et al	Tao et al	Vidić et al	Wang et al	Zu et al	Zheng et al
**Critical Appraisal**												
**Step 1: Are the results of the study valid?**												
1. Was the diagnostic test evaluated in a Representative spectrum of patients (like those in whom it would be used in practice)?	1	1	1	1	1	1	0	0	1	0	1	1
2.Was the reference standard applied regardless of the index test result?	1	1	1	1	1	1	1	0	0	1	0	1
3. Was there an independent, blind comparison between the index test and an appropriate reference ('gold') standard of diagnosis?	0	0	0	1	0	0	0	0	0	0	0	1
**Step 2: What were the results?**												
4.Are test characteristics presented?	0	3	2	3	0	3	1	3	1	1	1	3
**Step 3: Applicability of the results**												
5. Were the methods for performing the test described in sufficient detail to permit replication?	1	2	3	3	3	2	1	3	1	1	1	2
**Total score**	**3**	**7**	**7**	**9**	**5**	**7**	**3**	**6**	**3**	**3**	**3**	**8**
**Quality (%)**	**33.33333333**	**77.77777778**	**77.77777778**	**100**	**55.55555556**	**77.77777778**	**33.33333333**	**66.66666667**	**33.33333333**	**33.33333333**	**33.33333333**	**88.88888889**

Supplementary File 3

3: Critical Appraisal of Rectal studies

**Sl No**	**1**	**2**	**3**	**4**	**5**	**6**	**7**
**Author**	Bakke et al	Chen et al	Fusco et al	Li et al	Patrillo et al	Sun et al	Yang et al
**Critical Appraisal**							
**Step 1: Are the results of the study valid?**							
1. Was the diagnostic test evaluated in a Representative spectrum of patients (like those in whom it would be used in practice)?	1	1	1	1	1	1	1
2.Was the reference standard applied regardless of the index test result?	1	1	1	1	1	1	1
3. Was there an independent, blind comparison between the index test and an appropriate reference ('gold') standard of diagnosis?	0	0	1	0	0	0	0
**Step 2: What were the results?**							
4.Are test characteristics presented?	0	3	0	2	1	2	0
**Step 3: Applicability of the results**							
5. Were the methods for performing the test described in sufficient detail to permit replication?	3	2	0	3	0	3	3
**Total score**	**5**	**7**	**3**	**7**	**3**	**7**	**5**
**Quality (%)**	**55.55555556**	**77.77777778**	**33.33333333**	**77.77777778**	**33.33333333**	**77.77777778**	**55.55555556**
