# Supplementary material for: Diagnostic Efficacy and Correlation of Intravoxel Incoherent Motion (IVIM) and Contrast-Enhanced (CE) MRI Perfusion Parameters in Oncology Imaging: A Systematic Review and Meta-Analysis
Source: Int J Biomed Imaging. 2025 Nov 18;2025:3621023. doi: 10.1155/ijbi/3621023 (PMC12646736; doi:10.1155/ijbi/3621023)
Supplement: Supporting Information 3 — The quality assessment score process and scores of each study are in File S3. [file 3621023.f3.pdf]

Supplementary file 2

SEARCH STRATEGIES

Pubmed:

((((((("Meningioma"[Mesh]) OR "Glioma"[Mesh]) OR ("Breast Cancer Lymphedema"[Mesh] OR "Breast Neoplasms"[Mesh])) OR ("Rectal Neoplasms"[Mesh])) OR (("Carcinoma, Hepatocellular"[Mesh]) OR "Carcinoma, Renal Cell"[Mesh]) OR (("Liver Neoplasms"[Mesh])) AND (((((("IVIM") OR ("intravoxel Incoherent motion")) OR ("multi b value")) OR ("biexponential"))) OR ("Diffusion Magnetic Resonance Imaging"[Mesh]))) AND (((((("dynamic contrast enhanced") OR ("DCE")) OR ("dynamic susceptibility contrast")) OR ("DSC")) OR ("contrast-enhanced MR*")) OR ("multiparametric"))) AND (((((Efficacy) OR (Sensitivity)) OR (specificity)) OR (correlation)) OR (effectiveness))

Web of Science

ALL=((((((((Meningioma) OR Glioma) OR ("Breast Cancer Lymphedema" OR "Breast Neoplasms")) OR ("Rectal Neoplasms")) OR (("Carcinoma, Hepatocellular") OR "Carcinoma, Renal Cell")) AND ((((((IVIM) OR ("intravoxel Incoherent motion")) OR ("multi b value")) OR (biexponential))) OR ("Diffusion Magnetic Resonance Imaging"))) AND (((((("dynamic contrast enhanced") OR (DCE)) OR ("dynamic susceptibility contrast")) OR (DSC)) OR ("contrast--enhanced MR*")) OR (multiparametric))) AND (((((Efficacy) OR (Sensitivity)) OR (specificity)) OR (correlation)) OR (effectiveness)))

Embase:

('meningioma'/exp OR 'meningioma' OR 'glioma'/exp OR 'glioma' OR (('breast cancer lymphedema'/exp OR 'breast cancer lymphedema') OR ('breast neoplasms'/exp OR 'breast neoplasms')) OR 'rectal neoplasms'/exp OR 'rectal neoplasms' OR 'carcinoma, hepatocellular'/exp OR 'carcinoma, hepatocellular' OR 'carcinoma, renal cell'/exp OR 'carcinoma, renal cell') AND (ivim OR 'intravoxel incoherent motion'/exp OR 'intravoxel incoherent motion' OR 'multi b value' OR biexponential OR dwi) AND ('dynamic contrast enhanced'/exp OR 'dynamic contrast enhanced' OR dce OR 'dynamic susceptibility contrast'/exp OR 'dynamic susceptibility contrast' OR 'dsc'/exp OR dsc OR 'contrast--enhanced mr*' OR multiparametric) AND ('efficacy'/exp OR efficacy OR 'sensitivity'/exp OR sensitivity OR 'specificity'/exp OR specificity OR 'correlation'/exp OR correlation OR effectiveness) AND 'article'/it

Cochrane Library

((((((([mh Meningioma]) OR [mh Glioma]) OR ([mh "Breast Cancer Lymphedema"] OR [mh "Breast Neoplasms"])) OR ([mh "Rectal Neoplasms"])) OR (([mh "Carcinoma, Hepatocellular"]) OR [mh "Carcinoma, Renal Cell"]) OR (([mh "Liver Neoplasms"])) AND ((((((IVIM) OR ("intravoxel Incoherent motion")) OR ("multi b value")) OR (biexponential)) OR (DWI)))) AND (((((("dynamic contrast enhanced") OR (DCE)) OR ("dynamic susceptibility contrast")) OR (DSC)) OR (("contrast-enhanced" NEXT MR*))) OR (multiparametric))) AND (((((Efficacy) OR (Sensitivity)) OR (specificity)) OR (correlation)) OR (effectiveness)))

Scopus:

Supplementary file 2

(((((((INDEXTERMS (meningioma)) OR INDEXTERMS (glioma)) OR (INDEXTERMS ("breast cancer lymphedema") OR INDEXTERMS ("breast neoplasms"))) OR (INDEXTERMS ("rectal neoplasms"))) OR ((INDEXTERMS ("carcinoma, hepatocellular")) OR INDEXTERMS ("carcinoma, renal cell"))) AND ((((((TITLE-ABS-KEY (ivim)) OR (TITLE-ABS-KEY ("intravoxel incoherent motion"))) OR (TITLE-ABS-KEY ("multi b value"))) OR (TITLE-ABS-KEY (biexponential)))) OR (INDEXTERMS ("diffusion magnetic resonance imaging"))))) AND ((((((TITLE-ABS-KEY ("dynamic contrast enhanced")) OR (TITLE-ABS-KEY (dce))) OR (TITLE-ABS-KEY ("dynamic susceptibility contrast"))) OR (TITLE-ABS-KEY (dsc))) OR (TITLE-ABS-KEY ("contrast--enhanced mr*"))) OR (TITLE-ABS-KEY (multiparametric))))) AND (((((efficacy) OR (sensitivity)) OR (specificity)) OR (correlation)) OR (effectiveness))
